# A Rare Case of Moyamoya Disease in Infancy: Complexities in Post-stroke Neurorehabilitation

**DOI:** 10.7759/cureus.89133

**Published:** 2025-07-31

**Authors:** Amine Achraf Majit, Abdelilah Rhoul, Houssam Mahla, Khaoula Kassi, Ahmed Amine El Oumri

**Affiliations:** 1 Physical Medicine and Rehabilitation, Mohammed VI University Hospital, Oujda, MAR; 2 Physical Medicine and Rehabilitation, Faculty of Medicine and Pharmacy, Mohammed I University, Oujda, MAR

**Keywords:** brain vascular disease, cerebral ischemic stroke, infancy, moyamoya disease, neurorehabilitation, pediatric, pediatric rehabilitation, pediatric stroke, rehabilitation

## Abstract

Although ischemic stroke in infants is not very common, it remains one of the causes of morbidity and mortality in children. One of the etiologies is Moyamoya disease, which remains a rare entity in Morocco. Management of this disease in the pediatric population remains precarious due to delayed diagnosis, given the diversity of clinical presentations and the lack of necessary technical means, such as MRI or angiography, allowing early diagnosis, as well as the lack of standardized international rehabilitation protocols and the absence of pediatric rehabilitation structures in underdeveloped countries. Through this case report of a four-month-old infant who suffered an ischemic stroke in a Moyamoya, we discuss the diagnostic difficulties and its rehabilitation challenges.

## Introduction

Pediatric stroke shares the same definition as that of an adult stroke. However, a distinction is made based on the age of onset: perinatal stroke, which occurs between the 20th week of gestation and the 28th day of life, and infantile stroke, which affects children aged 28 days to 18 years. It is considered one of the 10 leading causes of infant mortality [1]. Several etiologies are involved. Moyamoya disease, a progressive cerebral arteriopathy characterized by stenosis and occlusion of the internal carotid artery, with the development of collateralization, whose angiographic appearance, a “cloud of smoke,” gives the disease its name. However, it is rarely responsible for stroke in infants, given its progressive nature [2]. We describe the case of a four-month-old infant who suffered a stroke due to moyamoya disease.

The diagnosis of stroke in children is often delayed, given the diversity of clinical presentation, particularly in infants (apnea, seizures, hypotonia). However, early neurological management and appropriate rehabilitation help reduce the disability caused by pediatric stroke and its impact on the child’s quality of life [3].

Even rehabilitation after pediatric stroke remains understudied. Developed countries have established guidelines for pediatric stroke, with specific recommendations, but these are unfortunately not adapted to low-income countries. This report aims to present a case of ischemic stroke due to Moyamoya disease, as well as current practices in infant motor rehabilitation, and to determine which of these techniques are applicable in a low-income country.

## Case presentation

A four-month-old boy presented with recurrent left-sided tonic-clonic seizures over the previous five days. On examination, the infant was conscious but minimally responsive. Neurological findings included left-sided hemiparesis, increased muscle tone, hyperreflexia more pronounced on the left side, and left facial paralysis.

**Figure 1:**
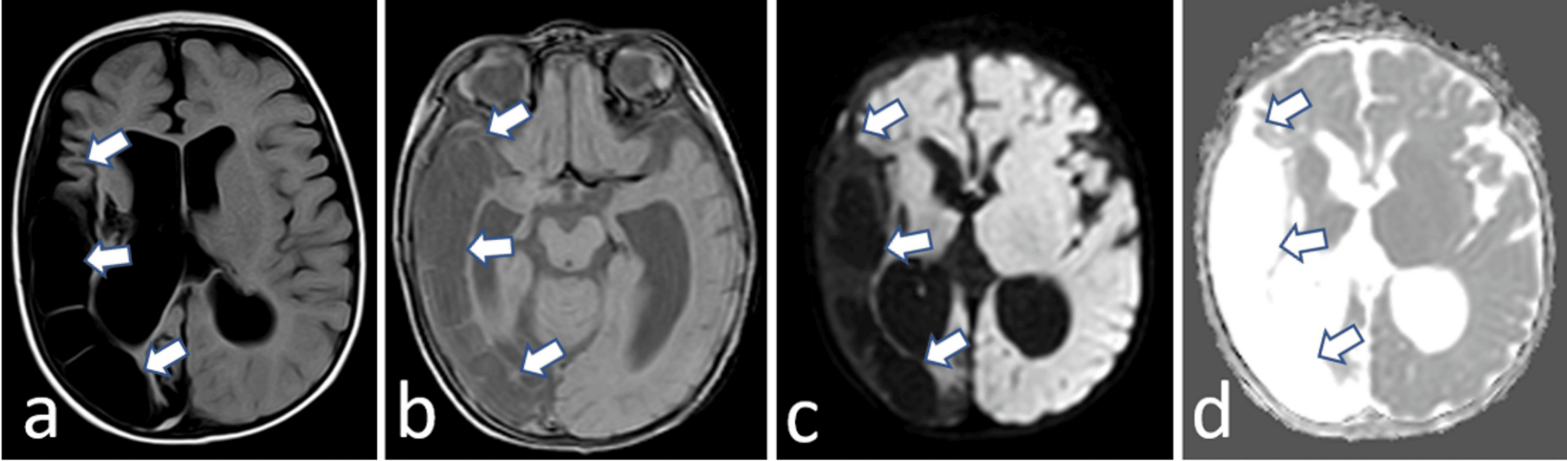
Brain MRI axial sequences. Fluid-attenuated inversion recovery (FLAIR) (a), SET1 (b), diffusion b1000 (c), and apparent diffusion coefficient (ADC) mapping (d). White arrows indicate a large right temporo-parieto-occipital area hypointense in FLAIR, T1, and diffusion, without diffusion restriction on ADC mapping. These findings are consistent with chronic stroke with resulting encephalomacia.

**Figure 2:**
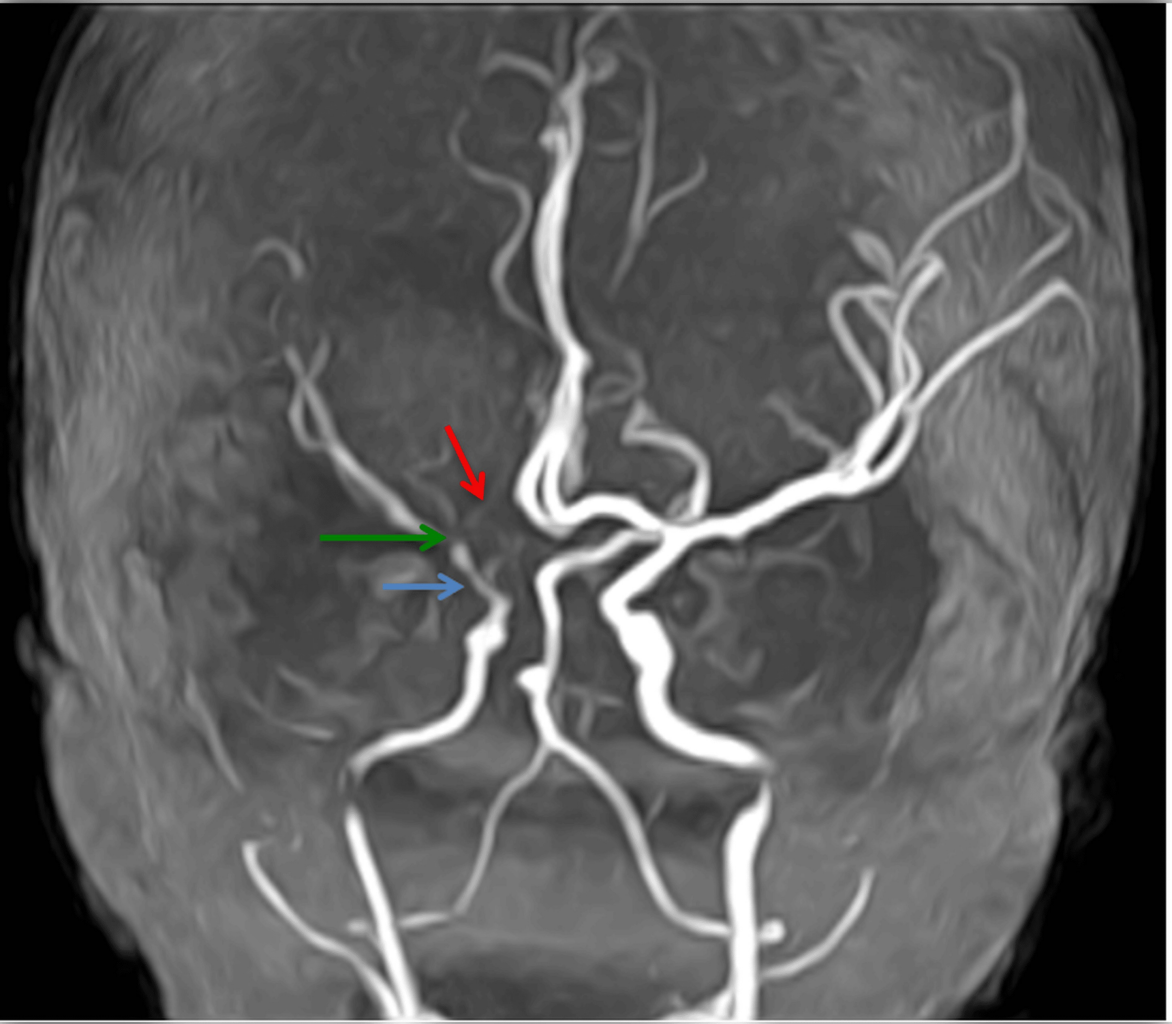
Three-dimensional time-of-flight sequence. Blue arrow: tight stenosis at the terminal portion of the right internal carotid artery. Green arrow: pre-occlusive stenosis of the right middle cerebral artery at its M1 portion. Red arrow: total occlusion of the A1 portion of the right anterior cerebral artery.

Cranial MRI and MR angiography (Figures 1, 2) revealed a macroscopic appearance of the right carotid artery in its petrous portion, occluded in its cavernous portion, with a macroscopic and irregular appearance of the ipsilateral Sylvian artery associated with engorgement of the distal arteries, suggestive of Moyamoya disease.

Our patient received neither thrombolysis nor thrombectomy. Due to the irreversible arterial occlusion, surgical revascularization was considered to enhance long-term collateral perfusion.

The etiological workup revealed no secondary causes and was normal. Acute management consisted of seizure control with levetiracetam, secondary stroke prevention with antiplatelet drugs (aspirin), and intensive rehabilitation sessions despite limited pediatric neurorehabilitation services.

Due to the limited number of available rehabilitation and psychomotor therapists, as well as the child’s mother’s inability to provide daily mobility, we implemented a program of three weekly physiotherapy and psychomotor therapy sessions. Despite this, compliance with the protocol was not satisfactorily maintained during the nine-month follow-up, during which a medical reassessment was performed every three months.

The nine-month assessment found a progressive improvement in axial tone, allowing incomplete cephalic control and a sitting position with unstable balance and postural asymmetry. Improvement in active mobility (abduction, antepulsion, elbow flexion) was noted. However, the child was not using his plegic hand and exhibited no bimanual coordination. He began to crawl awkwardly, as the muscle strength developed in his arms remained insufficient to support his weight (permanent contact of the abdomen with the ground), and without coordination of arm and leg movements. Progress was made in comprehension, attention, and social interaction, but, at this stage, there was an overall delay. The intensity and regularity of physiotherapy and psychomotor therapy sessions were essential to optimize the functional prognosis, but the child’s fatigue and the mother’s limited availability also posed an obstacle to effective rehabilitation.

## Discussion

The clinical presentation of ischemic stroke in infants is highly variable and nonspecific. Several symptoms can be noted, such as seizures, headaches, and hemiparesis. These symptoms, which may be present in other differential diagnoses, are the reason the diagnosis of stroke in children is often delayed [4]. The average time between the onset of symptoms and medical consultation is 20 hours [5]. Early neurological and rehabilitation management can reduce the disability caused by pediatric stroke and its impact on the child’s quality of life [3].

Infant strokes are often associated with underlying pathologies, i.e., heart disease, coagulation disorders, infectious and inflammatory diseases, and vascular causes. Moyamoya disease is a progressive cerebral arteriopathy associated with stenosis and occlusion of the intracranial internal carotid circulation, with the development of collateralization. It accounts for approximately one-fifth of cerebral arteriopathies identified in childhood strokes. The disease is classified as primary when idiopathic, and as secondary when associated with an underlying condition such as neurofibromatosis, Down syndrome, or prior cranial irradiation. However, the exact causes of Moyamoya disease remain unclear. Several etiologies have been proposed, including genetic, viral, traumatic, and iatrogenic [6].

Despite the presence of the *RNF213* gene, only imaging can confirm the diagnosis of Moyamoya disease [7,8]. Due to its accessibility and simplicity, regardless of the child’s clinical condition, brain CT, combined with CT angiography of the circle of Willis and perfusion CT, can identify early signs of cerebral ischemia and rule out hemorrhage. It can demonstrate or exclude the presence of proximal arterial occlusion, veno-sinus occlusion, ruptured cerebral vascular malformation, aneurysm, or arteriovenous malformation. In addition, crucial prognostic elements are identified, namely, volume of cerebral hemorrhage, ventricular flooding, acute hydrocephalus, mass effect, and cerebral commitments. The volume of cerebral infarction and the ischemic penumbra, obtained by perfusion CT, can guide therapeutic indications. However, the diversity of causes clinically simulating a stroke (stroke mimics) in children and the insufficient sensitivity of the scanner make MRI a more effective examination [9]. Although conventional cerebral angiography remains the most reliable method for staging the disease (Suzuki stages), it remains invasive; hence, MR angiography is very useful for diagnosis, vascular mapping, and monitoring [10].

Rehabilitation of motor disorders in infants and young children has been the subject of numerous studies, most of which focused on children with cerebral palsy (CP) [11]. However, CP encompasses a variety of pathologies. Specific rehabilitation after pediatric stroke remains understudied, although efforts are underway to extend some of the most effective motor recovery therapies to other areas, such as the restoration of sensory, language, attentional, and executive deficits. Most studies on rehabilitation of motor disorders have focused on the upper limbs, while few studies have examined the lower limbs. Mobilization should begin within the first few days of symptom onset, unless contraindicated [12].

Several rehabilitation techniques have been identified, and numerous studies have been conducted for each technique. Constraint-induced movement therapy (CIMT) involves restricting the healthy limb to promote the use of the injured side. Intensive hand-arm bimanual therapy (HABIT), which aims to improve function by using both hands together in everyday play and functional activities to facilitate coordinated movements of both limbs, has also been studied and shown promising results. It may offer similar benefits to motor cognitive therapy. CIMT results in similar improvements in hand function as HABIT. One potential advantage of bimanual training is that participants may make greater progress in achieving self-determined goals [12,13].

The combination of neuromodulation through transcranial direct current stimulation with CIMT or intensive occupational therapy has shown improvements in upper limb and hand motor function [11]. Similarly, the use of repetitive transcranial magnetic stimulation with CIMT has also been shown to be more effective than CIMT alone [11,13]. The association of botulinum toxin with modified CIMT has shown better results in terms of motor function than its combination with intensive conventional therapy [11,13]. The results of using neuromuscular electrical stimulation and functional electrical stimulation techniques have shown improvement in upper limb function [11,13].

Mirror therapy, which relies on the activation of mirror neurons in a specific area of ​​the motor cortex, was shown to be effective in improving upper limb activity, strength, motor speed, as well as precision and bilateral coordination [14], but was unfortunately not used in our case. Robotics and virtual reality are evolving technologies. Improvements in upper limb function after four weeks of using the ArmeoSpring (an exoskeleton that facilitates virtual play therapy through passive arm weight support), or the kidPEXO (another pediatric hand and arm exoskeleton), but they remain for children over three years old [15].

Numerous guidelines for pediatric stroke have been published by the American Heart Association, Canada, Australia, and the United Kingdom, offering specific rehabilitation suggestions. Unfortunately, these guidelines are not adapted to low-income countries, and rehabilitation management remains limited to these countries [11].

Although infant brain plasticity offers potential for recovery, ischemic damage can seriously compromise future development. Permanent neurological motor and cognitive impairments are observed in the majority of children who survive a stroke, even with early management and in specialized centers. Unlike the mature brain, some functions are not yet lateralized or specialized; healthy areas can potentially take over from damaged areas, which can limit permanent deficits; and immature neurons sometimes have better tolerance to lack of oxygen (adaptive mechanisms such as anaerobic glycolysis). Early neurological and rehabilitation management helps reduce the disability caused by pediatric stroke and its impact on the child’s quality of life [16].

## Conclusions

The low frequency of cerebral ischemic pathologies in newborns and infants, and their atypical clinical presentation, pose a real challenge for the management of this pathology. Several rehabilitation techniques have been developed for young children, but they remain unsuitable for infants. Each team has attempted to collaborate with its own series to find a consensus for better management, which is still being developed. Improvements have been observed in patient series owing to the combination of several rehabilitation techniques. However, these techniques remain unavailable in low-income countries despite their low cost.
